# Lymph Node Transplantation Decreases Swelling and Restores Immune Responses in a Transgenic Model of Lymphedema

**DOI:** 10.1371/journal.pone.0168259

**Published:** 2016-12-12

**Authors:** Jung-Ju Huang, Jason C. Gardenier, Geoffrey E. Hespe, Gabriela D. García Nores, Raghu P. Kataru, Catherine L. Ly, Inés Martínez-Corral, Sagrario Ortega, Babak J. Mehrara

**Affiliations:** 1 Department of Surgery, Division of Plastic and Reconstructive Surgery, Memorial Sloan Kettering Cancer Center, New York, New York, United States of America; 2 Department of Plastic and Reconstructive Surgery, Division of Reconstructive Microsurgery, Chang Gung Memorial Hospital, Chang Gung University, Taoyuan, Taiwan; 3 Department of Immunology, Genetics, and Pathology, Uppsala University, Uppsala, Sweden; 4 Transgenic Mice Unit, Biotechology Programme, Spanish National Cancer Research Centre, Madrid, Spain; USF Health Morsani College of Medicine, UNITED STATES

## Abstract

**Introduction:**

Secondary lymphedema is a common complication of cancer treatment and recent studies have demonstrated that lymph node transplantation (LNT) can decrease swelling, as well as the incidence of infections. However, although these results are exciting, the mechanisms by which LNT improves these pathologic findings of lymphedema remain unknown. Using a transgenic mouse model of lymphedema, this study sought to analyze the effect of LNT on lymphatic regeneration and T cell-mediated immune responses.

**Methods:**

We used a mouse model in which the expression of the human diphtheria toxin receptor is driven by the FLT4 promoter to enable the local ablation of the lymphatic system through subdermal hindlimb diphtheria toxin injections. Popliteal lymph node dissection was subsequently performed after a two-week recovery period, followed by either orthotopic LNT or sham surgery after an additional two weeks. Hindlimb swelling, lymphatic vessel regeneration, immune cell trafficking, and T cell-mediated immune responses were analyzed 10 weeks later.

**Results:**

LNT resulted in a marked decrease in hindlimb swelling, fibroadipose tissue deposition, and decreased accumulation of perilymphatic inflammatory cells, as compared to controls. In addition, LNT induced a marked lymphangiogenic response in both capillary and collecting lymphatic vessels. Interestingly, the resultant regenerated lymphatics were abnormal in appearance on lymphangiography, but LNT also led to a notable increase in dendritic cell trafficking from the periphery to the inguinal lymph nodes and improved adaptive immune responses.

**Conclusions:**

LNT decreases pathological changes of lymphedema and was shown to potently induce lymphangiogenesis. Lymphatic vessels induced by LNT were abnormal in appearance, but were functional and able to transport antigen-presenting cells. Animals treated with LNT have an increased ability to mount T cell-mediated immune responses when sensitized to antigens in the affected hindlimb.

## Introduction

Secondary lymphedema is a common complication of cancer treatment occurring in as many as 50% of patients who undergo lymph node dissection for breast cancer [1]. Although the extremities are most commonly affected, patients may also suffer from lymphedema in the region of the head, neck, or trunk depending on the precipitating factor [2, 3]. Regardless of the site, many patients with lymphedema suffer from functional issues such as heaviness, swelling, tightness, and pain, as well as skin infections that may require hospitalization [4, 5]. The current mainstay of lymphedema treatment is generally palliative, consisting of compression garments and physical therapy designed to relieve symptoms. This approach may be effective in select patients, but is limited by its time-consuming nature and high costs, often resulting in non-compliance and disease progression [6].

Recent advances in microsurgery have reignited research into the surgical treatment of lymphedema. Lymph node transplantation (LNT) is one promising approach in which healthy lymph nodes are harvested from a remote area and transplanted to the affected extremity using microsurgical techniques for revascularization [7–13]. In these cases, the lymphatic vessels are not repaired, but are thought to regenerate spontaneously as a result of a lymphangiogenic response [13, 14]. Several retrospective studies have reported promising outcomes with long-term symptomatic improvement, including improvement in limb swelling and decreased incidence of infections [8–13, 15–17]. Although the cellular mechanisms of this response remain unknown, it is thought that the transplanted lymph nodes shunt interstitial fluid to the systemic circulation through connections with the high endothelial venules and restore immune responses [14].

Although the clinical results of LNT for the treatment of lymphedema are exciting, this approach precludes systematic time course studies, tissue histological analysis, and mechanistic studies designed to identify the cellular mechanisms that regulate tissue changes. For example, it remains unknown whether LNT can reverse pathological skin changes such as hyperkeratosis or fibrosis. Similarly, while clinical studies suggest that lymphatic circulation is restored, it is unclear if regenerated lymphatics are capillary or collecting vessels. In addition, there is no evidence showing that these vessels connect with the transplanted lymph node or simply restore circulation to the next lymph node basin, or even if these vessels are physiologically functional and capable of transporting immune cells. Finally, although several authors have reported decreased incidence of cellulitis after LNT, it is unclear if this treatment has a direct effect on the impaired immune responses noted in lymphedema [17].

Over the past hundred years or so, a variety of animal surgical models of lymphedema have been reported [18–23]. Although these models have advanced our knowledge of lymphedema, they are imperfect, as they are limited by inconsistency in the degree of swelling, spontaneous resolution of lymphedema, and the need for extensive surgical (and often radiation) intervention with severe morbidity. In addition, the majority of previously described models do not match the temporal sequence of lymphedema clinically. The limb swelling that occurs in most of these models begins immediately after surgery and slowly resolves over time, whereas the clinical development of lymphedema usually occurs months, and often years, after the initial injury and is progressive once initiated [1]. This temporal sequence is important and suggests that additional tissue changes are necessary for the development of lymphedema and that these changes may not be adequately reflected by existing surgical models.

To overcome the shortcomings of the existing surgical models, we recently developed a non-surgical model of lymphedema that more closely matches the clinical presentation temporally, histologically, and radiologically [24]. In this model (FLT4-DTR), transgenic mice are created using Cre-Lox technology to drive the expression of human diphtheria toxin receptor (DTR) under the direction of FLT4 (the promoter for vascular endothelial growth factor receptor 3; VEGFR3). After DTR expression is activated using tamoxifen, subdermal administration of minute doses of diphtheria toxin (DT) in the hindlimb enables us to locally ablate the lymphatic system, resulting in chronic lymphedema that is similar to the clinical scenario. After DT injection, there is an immediate and marked swelling of the treated limb resulting from ablation of capillary and collecting lymphatics that partially resolves over the initial six-week period of time due to proliferation of the superficial lymphatic system. Chronic inflammatory responses, however, progressively impair lymphatic regeneration and function, leading to sustained severe swelling and fibrosis of the hindlimb that is present up to one year [24].

In the current study, we used our non-surgical model of lymphedema in conjunction with LNT to analyze the efficacy of this therapy in restoring lymphatic circulation and immune function. We report that LNT significantly improves the histopathologic changes of lymphedema by inducing formation of functional collateral lymphatic vessels that reconnect with the transplanted lymph node. This reconstituted lymphatic network, although abnormal in appearance, is capable of transporting antigen-presenting cells (APCs) to regional lymph nodes, leading to improved adaptive immune responses.

## Materials and Methods

### Animals and lymphatic ablation

All experimental protocols and procedures were approved by the Institutional Animal Care and Use Committee (IACUC) at Memorial Sloan Kettering Cancer Center, which operates under the Animal Welfare Act (AWA) and Health Research Extension Act of 1985. Animals were fed *ad lib* in a light- and temperature-controlled, pathogen-free animal care facility.

The FLT4-DTR model of non-surgical lymphedema has been previously reported and characterized [24]. Briefly, FLT4-CreER^T2^ mice (a gift from Dr. Sagrario Ortega, CNIO) were crossed with human DTR-floxed C57BL/6 mice (C57BL/6-Gt(ROSA)26Sor^tm1(HBEGF)Awai^/J (Jackson Laboratories, Bar Harbor, ME). FLT4 was selected as the target gene to allow for specific ablation of lymphatic endothelial cells (LECs) on both capillary and collecting lymphatic vessels. Genotyping confirmed the presence of double homozygous gene expression in the offspring prior to experimental use.

**Fig 1:**
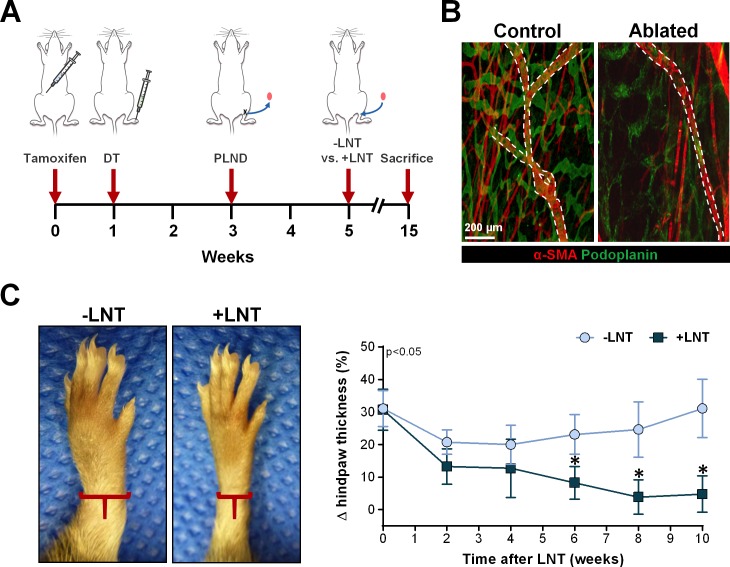
LNT decreases swelling after diphtheria toxin-induced lymphatic ablation. A) Schematic diagram of the experimental protocol. Transgenic mice underwent activation of the Cre-Lox recombination through a course of intraperitoneal injections of tamoxifen followed by a series of subdermal diphtheria toxin (DT) injections into the dorsum of the hindpaw one week later in order to ablate the local lymphatic system. Two weeks later, the mice underwent ipsilateral popliteal lymph node dissection (PLND). After an additional two week recovery, experimental mice underwent ipsilateral lymph node transplantation (+LNT), while control mice underwent popliteal incision without lymph node transplantation (-LNT). All analysis was done using hindlimbs of mice that were sacrificed 10 weeks after the final surgery. B) Representative whole-mount immunofluorescent image of the mouse hindlimb harvested one week after DT injection. Note ablation of both capillary (podoplanin^+^α-SMA^-^) and collecting lymphatics (podoplanin^+^α-SMA^+^). Collecting lymphatics are also indicated by the dotted white line. C) *Left panel*: Representative photograph of hindlimbs after surgery with or without LNT. Note the gross swelling in the hindlimb in the mouse without LNT (-LNT). *Right panel*: Quantification of change in hindpaw thickness from baseline in the weeks following surgery with or without LNT. Note significant decrease in mice that underwent LNT (+LNT) beginning 2 weeks after surgery.

Transgenic mice aged 8–12 weeks underwent intraperitoneal injections of 300 mg/kg of tamoxifen every other day for a total of 5 doses (**Fig 1A**). One week after activation, lymphedema of the hindlimb was induced by subdermal DT injection into the dorsal hindpaw (5 ng/day for 3 days). Two weeks after lymphatic ablation, animals underwent popliteal lymph node dissection (PLND) using a modification of previously reported methods [25]. Briefly, a transverse 0.5 cm incision was made over the popliteal area and the popliteal lymph nodes together with the surrounding fat were directly visualized and surgically excised. After a two-week recovery, animals were randomized to LNT or sham surgery using a modification of our previously reported methods [14]. In experimental animals, the previously used incision was opened and a popliteal lymph node graft harvested from a syngeneic wild-type donor mouse (C57BL/6; Jackson Laboratories, Bar Harbor, ME) was transplanted in the popliteal fossa followed by primary wound closure. In sham surgery, the incision was reopened, but no LNT was performed and the incision was closed similarly. Animals were sacrificed 10 weeks after surgery with or without LNT.

### Lymphedema measurements, immunohistochemistry, and histologic analysis

Hindpaw thickness was measured weekly in standardized locations (2 mm proximal to the tarsal joint) using a digital caliber by two blinded reviewers and swelling was quantified as a percentage increase from baseline (i.e., prior to lymphatic ablation). Histological cross-sections were harvested from the same area, fixed using 4% paraformaldehyde (PFA; Affymetrix, Cleveland, OH) for 48 hours, decalcified using 0.5 M sodium ethylenediaminetetraacetic acid (EDTA; Sigma-Aldrich, St. Louis, MO) for 72 hours, and embedded in paraffin. Lymph nodes and ear skin were fixed using 4% PFA for 12 hours and embedded in paraffin without decalcification. All tissues were analyzed using 5 μm sections.

Immunohistochemical staining was performed using our previously reported methods [26]. Briefly, paraffin-embedded tissues were rehydrated and antigen retrieval was performed using boiling sodium citrate (Sigma-Aldrich, St. Louis, MO). Endogenous peroxidase activity was quenched and nonspecific binding was blocked with a solution of 20% donkey serum (Sigma-Aldrich, St. Louis, MO) and 80% phosphate-buffered saline (PBS; Thermo-Fisher Scientific, Waltham, MA) for 1 hour in room temperature. Tissues were then incubated followed at 4°C with primary antibody overnight. The following primary antibodies were used: monoclonal anti-lymphatic vessel endothelial hyaluronan-1 (LYVE-1), anti-CD45 (R&D, Minneapolis, MN); anti-alpha-smooth muscle actin (α-SMA; Sigma-Aldrich, St. Louis, MO); anti-CD3 (Dako, Carpinteria, CA); anti-podoplanin, and anti-collagen I (Abcam, Cambridge, MA). After washing with PBS (Thermo-Fisher Scientific, Waltham, MA), sections were then incubated with the corresponding fluorescent-labeled secondary antibody conjugates (Life Technologies, Carlsbad, CA) for 5 hours. The sections were mounted using Mowiol (Sigma-Aldrich, St. Louis, MO).

Sections were scanned using a Mirax slide scanner (Zeiss, Munich, Germany) and analyzed with brightfield for hematoxylin and eosin (H&E) staining or fluorescent microscopy for immunofluorescent staining. The images were viewed and analyzed using Pannoramic Viewer (3DHistech, Budapest, Hungary). Fibroadipose deposition area was quantified as a percentage of the total tissue area in the cross-section. Dermal, epidermal, and α-SMA thickness were measured at the points of greatest thickness in each cross-section. Similarly, greatest diameter for each identified collecting lymphatic was recorded. The number of collecting lymphatics was quantified by counting the number of vessels in standardized areas measuring 0.25 mm^2^ with analysis of 4 sections per animal in each group. Type I collagen deposition in the dermis and subcutaneous tissue was assessed as a ratio of the area of positively stained dermis without a fixed threshold to total tissue area. In contrast, perilymphatic inflammatory cell infiltration was quantified by counting the number of inflammatory cells located within 50 μm of lymphatic vessel under 40x magnification and analysis of 4 sections per animal in each group. A total of 6–8 mice were analyzed in each group. For analysis of high powered fields (HPFs), the sections were reviewed in a blinded manner with a minimum of 3 HPFs per animal.

### Lymphangiography

Indocyanine green (ICG) lymphangiography was performed as previously described 10 weeks after surgery with or without LNT [27, 28]. Briefly, 15 μL of ICG (150 μg/mL diluted in sterile water) (Sigma-Aldrich, St. Louis, MO) was injected intradermally into the dorsal aspect of the hindpaw with the mice under brief sedation with 1.5% isoflurane inhalation (Henry Schein, Dublin, OH). After injection, the mice were allowed to move freely for 30 minutes, after which they were re-anesthetized for experimental analysis. The mice were placed on a heating pad and hindlimb lymphatics were visualized in a dark room using a custom-made EVOS EMCCD camera (Life Technologies, Carlsbad, CA) with an LED light source (CoolLED, Andover, UK) for 30 minutes. Static images were obtained using a Zeiss SteREO Lumar microscope V12 (Caliper Life Science, Hopington, MA).

### Analysis of immune function

Dendritic cell (DC) migration was assessed 10 weeks after surgery with or without LNT using a modification of previously reported methods [29]. Briefly, the ipsilateral hindlimbs were painted with 20 μl of 8% fluorescein isothiocyanate (FITC) isomer I (5 mg/mL; Sigma-Aldrich) in a 1:1 mixture of dibutylphalate and acetone (both from Sigma-Aldrich, St. Louis, MO). Eighteen hours later, the mice were sacrificed and the ipsilateral inguinal lymph nodes were harvested and digested to obtain single-cell suspensions. Migrating DCs were analyzed using flow cytometry (LSR II flow cytometer, BD Biosciences, San Diego, CA) with gating for FITC^+^/CD11c^+^ cells through FlowJo Software (Tree Star, Ashland OR).

To study T cell-mediated immune responses, we used a well-described model of contact hypersensitivity in which T cell responses are elicited at a remote site after sensitization with dinitrofluorobenzene (DNFB; Toronto Research Chemicals Inc., Toronto, Ontario) [30, 31]. Briefly, experimental and control mice were sensitized with 25 μl of 0.5% in acetone with 20% olive oil applied topically to the distal ipsilateral hindlimbs. Five days later, we challenged the contralateral ear with 0.3% DNFB. The mice were then sacrificed 3 days later for histological analysis of ear inflammation.

### Statistical analysis

Statistical analysis was performed using GraphPad Prism software (GraphPad Software, Inc., San Diego, CA). Comparisons between two groups were performed using the Student’s T test, while analysis of variance (ANOVA) with *post-hoc* tests was utilized for group comparisons. Data are presented as mean ± standard deviation unless otherwise noted and p<0.05 was considered significant.

## Results

### LNT decreases pathological changes of lymphedema

Consistent with our prior studies, DT injection in the hindlimb of FLT4-DTR mice resulted in local ablation of capillary and collecting lymphatics (**Fig 1B**) [24]. Similar to the temporal pattern of clinical lymphedema, these mice had an initial spike in limb swelling that decreased over the initial few weeks only to increase progressively over the following weeks, as reflected by the 31.1% increase in hindlimb thickness noted at 10 weeks after surgery without LNT in control mice (**Fig 1C**). In contrast, mice treated with LNT after lymphatic ablation had only a 4.8% increase in hindlimb thickness from baseline with an essentially normal hindlimb appearance. We also found that hindlimb swelling progressively decreased after LNT and became significantly different from that in mice without LNT starting at 2 weeks after transplantation (p = 0.017).

**Fig 2:**
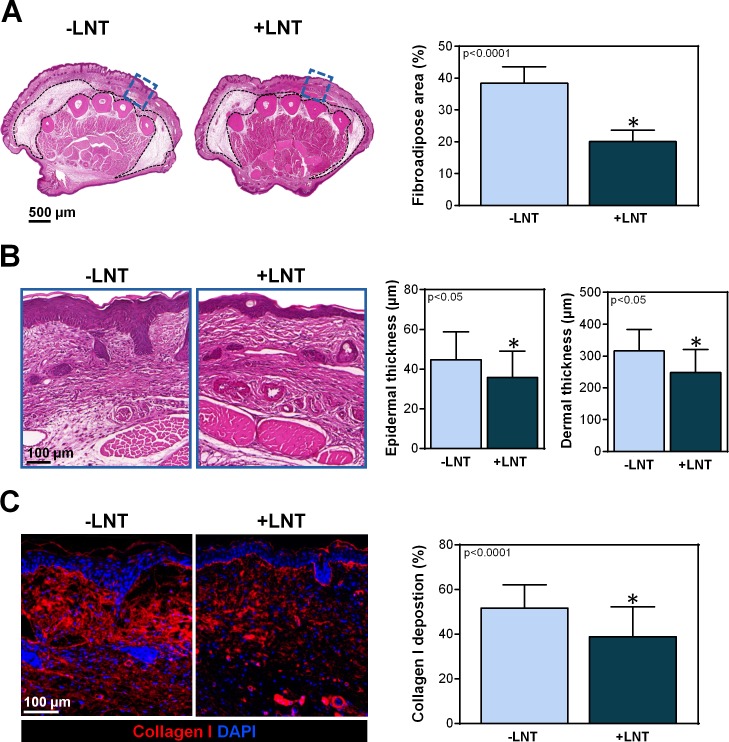
LNT decreases the pathological changes of lymphedema. A) *Left panel*: Representative H&E stain of the hindlimbs of mice with or without LNT. Cross-sections were obtained 2 mm proximal to the tarsal joint. The dotted black line indicates the area of fibroadipose deposition. The area highlighted by the blue dotted box is shown in high-power view in part B. *Right panel*: Quantification of the percentage of fibroadipose deposition area of hindlimbs of mice with and without LNT. B) *Left panel*: Representative high-power view of the areas indicated in the blue dotted boxes in part A. Note the decreased hyperkeratosis and dermal thickness in mice treated with LNT (+LNT). *Right panel*: Quantification of epidermal and dermal thickness in hindlimbs of mice with and without LNT. C) *Left panel*: Representative immunofluorescent images of hindlimbs stained for type I collagen (red) and nuclear DAPI (blue). Note decreased type I collagen deposition in mice treated with LNT (+LNT). *Right panel*: Quantification of type I collagen deposition (measured as a percentage of the total slide stained area) after surgery with and without LNT.

Lymphedema is clinically characterized by swelling, fibroadipose deposition, hyperkeratosis, and fibrosis [32]. As expected, the same findings were noted following lymphatic depletion in the transgenic mice without LNT (**Fig 2A–2C**). In contrast, mice treated with LNT 2 weeks after PLND had significantly less fibroadipose deposition (20.1 ± 1.47 vs. 38.4 ± 1.83% of cross-sectional area; p<0.001), hyperkeratosis (35.8 ± 3.22 vs. 44.7 ± 2.88 μm; p<0.05), dermal thickness (248 ± 73 vs. 316 ± 65 μm; p<0.05), and type I collagen deposition (38.9 ± 2.23 vs. 51.7 ± 1.42%; p<0.0001) (**Fig 2A–2C**). Taken together with our previous time-course study of changes in FLT4-DTR mice showing that fibroadipose deposition, hyperkeratosis, and fibrosis are significantly increased from baseline as early as 3 weeks after DT injection, these findings suggest that LNT reverses, rather than prevents, these pathologic skin changes [24].

### LNT decreases perilymphatic accumulation of inflammatory cells

**Fig 3:**
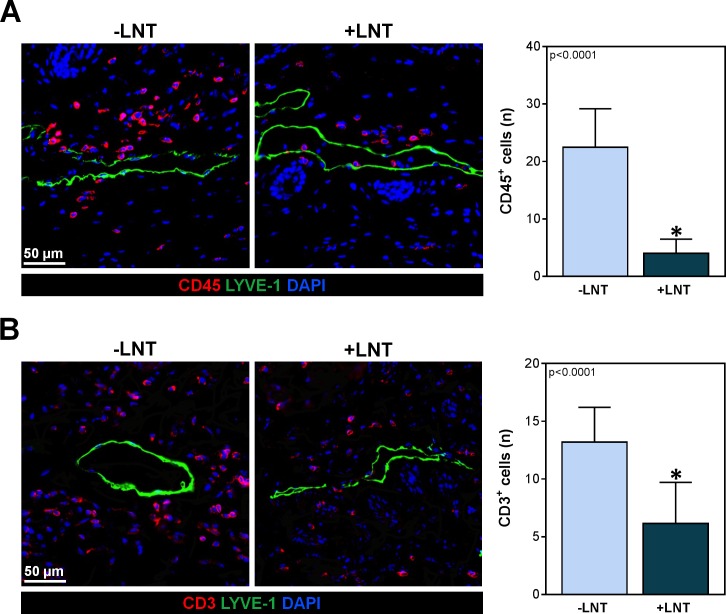
LNT decreases perilymphatic accumulation of inflammatory cells. A) *Left panel*: Representative images of CD45^+^ cells (red) surrounding subdermal lymphatic vessels (green) in hindlimbs of mice with or without LNT. *Right panel*: Quantification of CD45^+^ cells located within a 50 μm radius of lymphatic vessels in the hindlimbs of mice with and without LNT. B) *Left panel*: Representative images of CD3^+^ cells (red) surrounding the subdermal lymphatic vessels (green) in hindlimbs of mice in the hindlimbs of mice with and without LNT. *Right panel*: Quantification of perilymphatic CD3^+^ cells located within a 50 μm radius of lymphatic vessels in the hindlimbs of mice with and without LNT.

A number of studies have shown that T cells play a key role in the development of chronic skin and cutaneous tissue inflammation in lymphedema. In addition, we have found that inflammatory cells tend to cluster around lymphatic vessels within a 50 μm area rather than in a random distribution in the subcutaneous tissues [32]. Consistent with this, we noted that control mice that did not have LNT following PLND had a marked accumulation of CD45^+^ and CD3^+^ T cells around capillary lymphatics just below the dermis (**Fig 3A and 3B**). On the other hand, animals treated with LNT had a significant decrease in this inflammatory response resulting in fewer leukocytes overall (4.0 ± 2.5 vs. 22.5 ± 6.7 CD45^+^ cells/HPF; p<0.0001) and T cells in particular (6.2 ± 3.6 vs. 13.2 ± 3.1 CD3^+^ cells/HPF; p<0.0001).

### LNT promotes regeneration of functional lymphatic vessels

**Fig 4:**
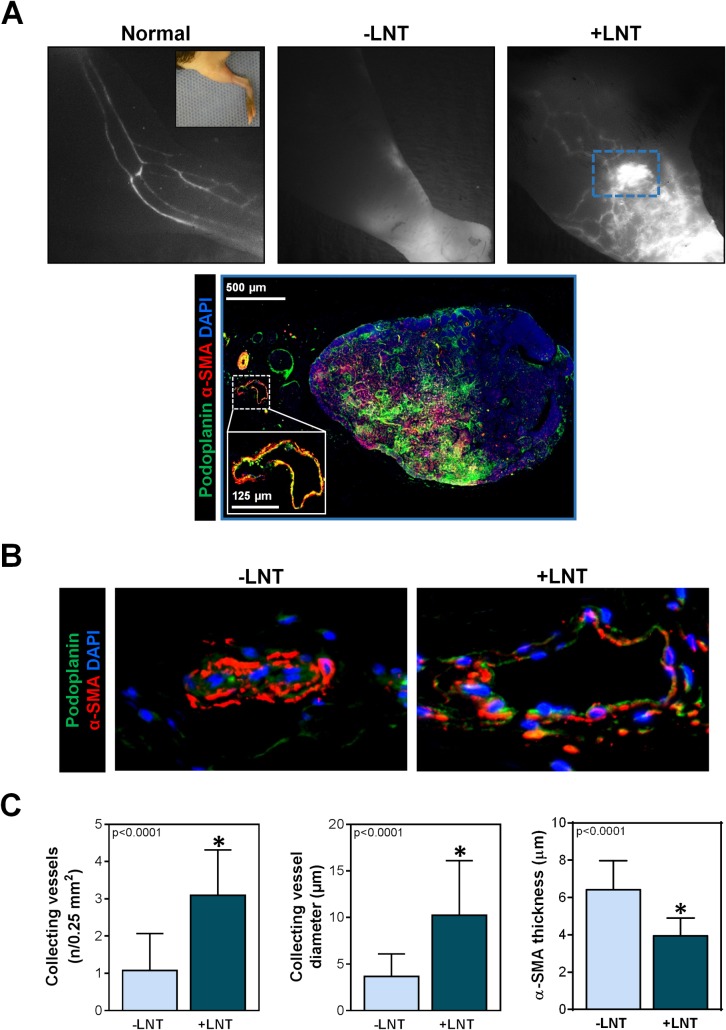
LNT promotes regeneration of collecting lymphatic vessels. A) *Upper panel*: Representative images of ICG lymphangiograms of normal mice (i.e., Cre-Lox mice without DT ablation), mice that had undergone local DT ablation followed by PLND but no LNT (-LNT), and mice that had undergone local DT ablation followed by PLND then LNT (+LNT). Main lymphatic collecting vessels can be seen in the normal hindlimb as white parallel linear structures, whereas no visible collecting vessels are noted in mice with ablated lymphatic circulation and no LNT (-LNT). Also note the dermal reflux as represented by accumulation of white dye diffusely in the mice without LNT (-LNT). In contrast, mice treated with LNT after lymphatic ablation (+LNT) have abnormal hyperplastic lymphatic vessels that converging toward the transplanted node, as indicated by the bright white spot in the blue dotted box. *Lower panel*: Immunofluorescent image of the transplanted lymph node indicated by the blue dotted box in the upper panel. Lymphatic collecting vessels indicated by podoplanin (green) and α-SMA (red). The inset in the lower left corner represents the magnified view of the lymphatic vessel adjacent to the transplant lymph node as indicated by the white dotted line. B) Representative immunofluorescent images of collecting lymphatics in mice with and without LNT. Note the collapsed lumen and proliferation of α-SMA^+^ cells in mice without LNT (-LNT). *C)* Quantification of collecting vessels in a 0.25 mm^2^ area, collecting vessel diameter, and α-SMA thickness in mice with and without LNT. Note the significant increase in number of collecting vessels and luminal diameter with a corresponding decrease in α-SMA thickness in mice treated with LNT (+LNT).

Similar to clinical findings of lymphedema, ICG lymphangiography of mice without LNT demonstrated dermal reflux of interstitial fluid and the absence of functional lymphatic vessels (**Fig 4A**). In contrast, mice treated with LNT had a large number of tortuous lymphatic vessels that connected to the transplanted lymph node. Although these lymphatic vessels appeared distinctly different than collecting lymphatics of a normal hindlimb, they contained ICG, indicating the presence of lymphatic flow. In addition, although mice treated with LNT also had mild dermal reflux (as indicated by diffuse white staining in **Fig 4A, right upper panel**), this was less than that observed in mice without LNT. Furthermore, histological examination of transplanted lymph nodes and the surrounding structures revealed collecting lymphatics, as indicated by podoplanin^+^ cells surrounded by with α-SMA^+^ cells, directly adjacent to the lymph node, thereby suggesting that afferent or efferent vessels had reconnected with the transplanted lymph node. Interestingly, collecting lymphatics were histologically present in mice with and without LNT, but, similar to clinical findings of late-stage lymphedema, the few collecting vessels in mice without LNT were nearly completely sclerosed and collapsed with a thick surrounding layer of **α**-SMA^+^ cells (**Fig 4B and 4C**) [33]. In contrast, mice that had undergone LNT had a significantly increased number of collecting lymphatics. These vessels were tortuous, but the mean diameter was more than 2.5-fold greater (10.2 ± 5.9 vs. 3.6 ± 2.5 μm; p<0.001) with a thinner **α**-SMA layer (3.9 ± 1.0 vs. 6.5 ± 1.6 μm; p<0.0001), as compared to that of control mice. Taken together, these findings suggest that LNT promotes regeneration of functional lymphatic vessels and decreases pathologic changes in lymphatic collecting vessels.

### LNT increases DC trafficking and T cell-mediated responses

**Fig 5:**
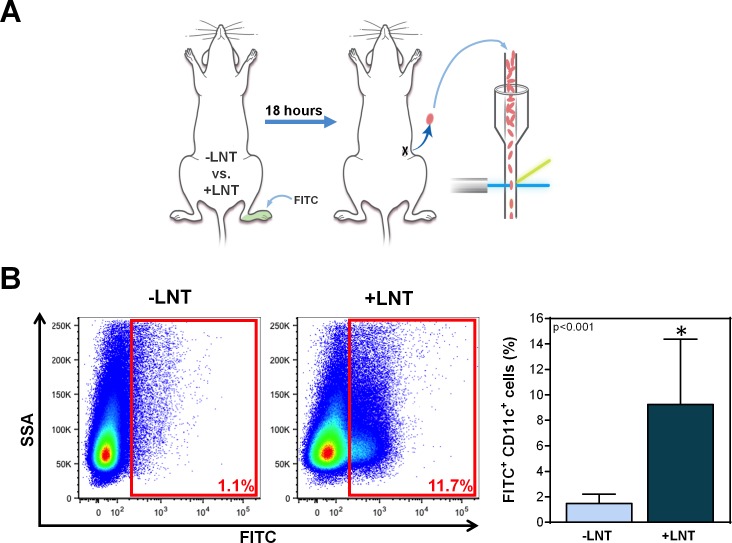
LNT increases migration of DCs to inguinal lymph node. A) Schematic diagram of the experimental protocol. Ten weeks after surgery with or without LNT, mice underwent FITC painting of the ipsilateral distal hindlimb. Eighteen hours later, the number of DCs migrating to the inguinal lymph node was analyzed using flow cytometry. B) *Left panel*: Representative flow cytometry plots of inguinal lymph node cells gated for side scatter analysis (SSA) and FITC. The red boxes indicate the gating for FITC^+^ DCs. *Right panel*: Quantification of FITC^+^ DCs in the inguinal lymph nodes of mice treated with or without LNT.

DC trafficking to regional lymph nodes via lymphatic channels is a key step in antigen presentation and activation of adaptive immune responses [34]. Consistent with our ICG lymphangiography studies, we found that mice treated with LNT had significantly increased trafficking of DCs to the inguinal lymph node (the next lymph node in the chain after the popliteal lymph node), as assessed by FITC painting of the distal hindlimb (**Fig 5A and 5B**). In fact, LNT resulted in a 6-fold increase in trafficked DCs in the inguinal lymph node compared to control mice without LNT (9.2 ± 5.1 vs. 1.5 ± 0.7% of cells; p<0.001).

**Fig 6:**
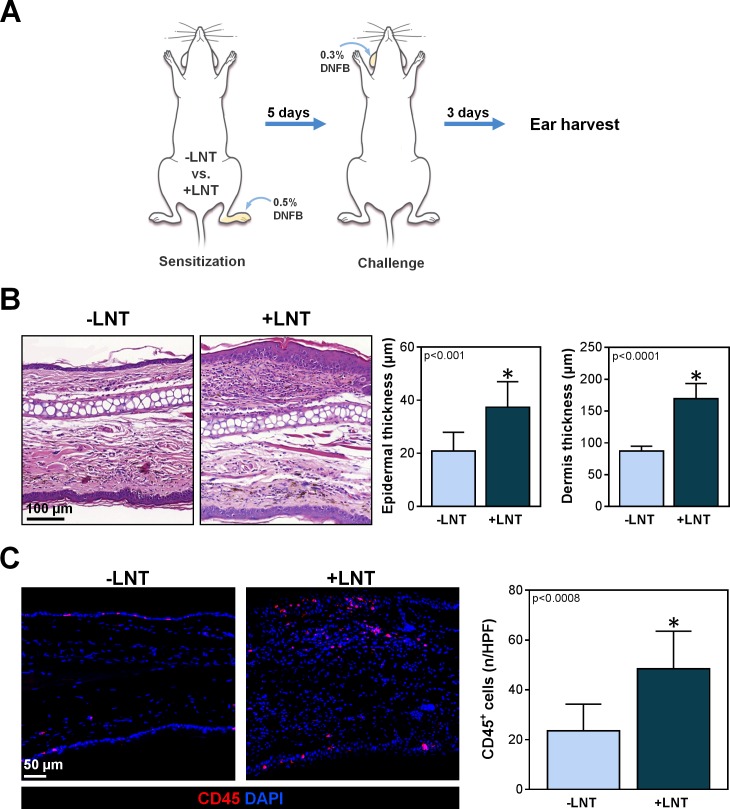
LNT improves T cell-mediated immune responses. A) Schematic diagram of experimental protocol. Ten weeks after surgery with or without LNT, mice were sensitized with topical 0.5% DNFB to the ipsilateral hindlimb once daily for three days. T cell response was elicited by challenging the contralateral ear with topical 0.3% DNFB 5 days later. The ears were harvested for analysis 3 days later. B) *Left panel*: Representative H&E stain of the ear skin from mice with and without LNT. Note the increased inflammatory reaction in mice treated with LNT (+LNT). *Right panel*: Quantification of epidermal and dermal thickness of ear skin. Note significant increase in both epidermal and dermal thickness in mice treated with LNT (+LNT). C) *Left panel*: Representative images demonstrating immunofluorescent localization of CD45^+^ cells (red) in ear skin of mice with and without LNT. *Right panel*: Quantification of CD45^+^ cells per HPF (80x) in mice treated with and without LNT. Note the more robust inflammatory response as indicated by the greater amount of CD45^+^ cells in mice treated with LNT (+LNT).

To determine if increased DC trafficking in LNT mice corresponded with improved T cell-mediated responses, we performed a standard assay of T cell activation with DNFB sensitization (**Fig 6A**) [34]. Compared to control animals, mice treated with LNT had a significant increase in T cell response as indicated by increased epidermal thickness (37.4 ± 9.6 vs. 20.1 ± 7 μm; p<0.001), dermal thickness (170 ± 23.7 vs. 87.1 ± 7.7 μm; p<0.001), and infiltration of leukocytes (48.4 ± 15.2 vs. 23.5 ± 10.7 CD45^+^ cells/HPF; p<0.001) (**Fig 6B and 6C**). Taken together, these findings suggests that LNT can lead to improvement in impaired adaptive immunity due to lymphatic injury through increased DC trafficking to regional lymph nodes and greater T cell-mediated responses.

## Discussion

Using a novel FLT4-DTR mouse model of lymphedema, we found that LNT after lymphatic injury leads to reversal of several key pathologic features of lymphedema, including swelling, skin changes, and local immunosuppression. Similar to clinical studies, these improvements can likely be attributed to the regeneration of lymphatic collecting vessels from preexisting lymphatic vessels [11, 24, 35]. Based on our prior studies, we have found that DT injection ablates 60–80% of LECs depending on the dose of DT utilized and that superficial lymphatics distal to the zone of injection are likely spared, therefore leaving behind lymphatic vessels that can proliferate in response to lymphangiogenic signals [24]. Furthermore, successful connection of the collateral lymphatic vessels to the transplanted lymph nodes then allow the lymph nodes to separate the lymphatic system into prenodal and postnodal pressure areas, thereby creating a more optimal environment for capillary vessel pumping and eventual differentiation into functional collecting vessels [18, 35]. Although we noted that the regenerated vessels following LNT were abnormal in appearance, they were functional, as indicated by their ability to transport dye and APCs. In addition, increased DC trafficking following LNT consequently led to restoration of T cell-mediated immune responses through improved antigen presentation and memory T cell generation.

Adipogenesis, inflammation, and fibrosis are all clinical hallmarks of lymphedema [36, 37]. In fact, end-stage lymphedema becomes resistant to conventional compression and massage therapy when interstitial fluid is replaced by progressive fibroadipose tissue deposition [6]. In addition, other studies utilizing transgenic mouse models of lymphedema have shown that leaky lymphatic vessels promote adipose cell proliferation [38]. In the current study, we have shown for the first time that these pathological skin changes of lymphedema are reversible with LNT in a mouse model. Our findings are supported by our prior clinical study demonstrating that skin changes in patients with unilateral upper extremity lymphedema are improved with lymphaticovenous bypass, a procedure in which lymphatic vessels are microsurgically anastomosed to the venous circulation to bypass the zone of obstruction [39]. It is likely that physiologic surgical procedures that decrease lymphatic stasis, such as LNT and lymphaticovenous bypass, reverse the pathology of lymphedema by decreasing the driving stimulus of this disease. This is in contrast to surgical interventions that only treat the end result through excision or removal of the fibrofatty tissues (i.e., liposuction); such procedures simply treat the symptoms rather than curing the underlying pathology.

As previously mentioned, our findings are consistent with our prior experiments in which LNT in an axillary lymph node dissection mouse model results in the expression of lymphangiogenic growth factors in the transplanted lymph node, as well as in the perinodal fat, resulting in lymphatic vessels that spontaneously reconnect to surrounding lymphatics after transplantation [14, 40]. Similarly, other reports have demonstrated that exogenously applied vascular endothelial growth factor C (VEGF-C) increases lymph node lymphangiogenesis in porcine and mouse models of lymphadenectomy [13, 35, 41]. Our results are particularly important because these prior studies were performed using models of lymphadenectomy rather than true lymphedema and may not reflect the clinical pathology as well as our FLT4-DTR model of lymphatic ablation. Because lymphangiogenesis is thought to occur as a balance between pro- and anti-lymphangiogenic forces, future efforts will be needed to delineate the role of the individual forces regulating lymphangiogenesis after LNT [42–44].

In our previous studies, we have also found that the architecture of transferred lymph node is preserved after LNT (i.e., maintenance of T and B cell zones) [40]. Building upon this, our current study revealed that transplanted lymph nodes enabled DC migration to downstream lymph node basins and led to restoration of T cell-mediated immune responses, as evidenced by the greater inflammatory response in LNT mice after DNFB sensitization and challenge compared to mice without LNT (DNFB sensitivity is mediated by CD8^+^ cells). The fact that DCs migrated to the inguinal lymph node and sensitized T cell responses provides further evidence that lymphatics regenerated by LNT are functional since a major function of the lymphatic system is to transport immune cells. This hypothesis is also supported by our finding that animals with LNT had decreased accumulation of perilymphatic inflammatory cells, likely because the functional lymphatics provided a route of exit for these cells [45]. This is clinically relevant, as recurrent infections and anergy in the lymphedematous limb comprise a significant morbidity in lymphedema. The effect of LNT on innate immune responses remains unclear and is currently under investigation.

As previously noted, the rise of LNT as a treatment option for lymphedema corresponded with the development of microsurgical techniques utilized for lymph node revascularization. It is important to note that the LNT in this study does not include revascularization, as microsurgical anastomosis is technically challenging in a mouse model and would likely lead to greater morbidity. However, as we have shown in our prior studies, the small size of the transplanted murine lymph node enables spontaneous revascularization without evidence of necrosis or ischemia 2 or 4 weeks after transplantation [14, 40]. This is consistent with the use of composite grafts clinically, in which grafts measuring less than 1.5 cm have rapid revascularization [46]. Consequently, while we cannot prove conclusively that short-term ischemia in the transplanted lymph node does not have an effect, it seems unlikely based on our current and prior findings. As such, the mouse model is actually a particularly useful model with which to specifically study lymphangiogenesis independent of revascularization. Of note, multiple studies in larger animal models such as sheep, dogs, and pigs have shown that revascularization increases lymph node survival and lymphatic function [18, 47]. However, many of these studies utilized models of lymphatic injury that do not correlate to the clinical scenario as well as our lymphatic ablation model. In addition, some of these studies are limited by the absence of clinical lymphedema prior to transplantation, so the results may be confounded with less applicable clinical translation [47]. Therefore, although clinical studies provide evidence for the benefit of revascularization, future studies in larger animal models that closely simulate lymphedema are necessary to understand the cellular mechanisms underlying the effect of vascularized LNT on lymphatic function [12].

In conclusion, LNT in a transgenic mouse model of lymphedema leads to reversal of key pathologic changes of lymphedema by promoting the regeneration of functional lymphatic collateral vessels. Although abnormal in appearance, the regenerated lymphatic vessels are able to prevent lymphatic fluid stasis and transport macromolecules. In doing so, LNT leads to improvement in swelling, skin changes such as hyperkeratosis and fibrosis, and T cell-mediated immune responses. These findings are important and clinically relevant, especially as LNT becomes increasingly popular as a surgical treatment for lymphedema. This new understanding of the cellular mechanisms through which LNT works may also provide insight into treatments that may increase efficacy or identify patients who would be most likely to respond.
